# Challenges and Outlook of Veterinary Education in Iran: A Survey of Veterinary Students at Urmia University During the COVID‐19 Pandemic

**DOI:** 10.1002/vms3.70081

**Published:** 2025-01-20

**Authors:** Mojtaba Hadian, Bahram Dalir‐Naghadeh

**Affiliations:** ^1^ Department of Internal Medicine and Clinical Pathology Faculty of Veterinary Medicine Urmia University Urmia Iran

**Keywords:** career preferences, Covid‐19, Iran, students' satisfaction, veterinary education

## Abstract

**Background:**

Veterinary science remains a challenging and less appreciated subject in the Iranian higher education system and society.

**Objectives:**

This survey aimed to assess the satisfaction and outlook of veterinary students at Urmia University, one of the major veterinary faculties in Iran. The survey was conducted during the COVID‐19 pandemic, which also significantly impacted veterinary education.

**Methods:**

A questionnaire was used to gather the opinions of 292 students.

**Results:**

The results revealed that 50.2% of respondents were either completely unsatisfied or less satisfied with the quality of teaching. Poor clinical training, the curriculum and distance learning were identified as the primary reasons. In addition, 40.3% of students expressed a desire to change their major. Most students (82.6%) expressed a desire to continue their education after completing their undergraduate course. Regarding future employment, 45.8% of participants wanted to start their own business or practice, whereas 54.2% preferred employment opportunities. A significant 81.6% of participants said they would consider emigrating to pursue better career prospects.

**Conclusion:**

The study highlighted various challenges in veterinary education in Iran, including dissatisfaction with the quality of teaching, an oversupply of trained veterinarians and limited career options. The COVID‐19 pandemic and distance learning further exacerbated these issues.

## Graphical abstract

The survey revealed significant dissatisfaction, with 50.2% unhappy with teaching quality, citing poor clinical training and distance learning as key issues. In addition, 40.3% considered changing majors, whereas 82.6% wished to pursue further education. Many students expressed entrepreneurial aspirations or preferred employment, and 81.6% would consider emigrating for better opportunities.

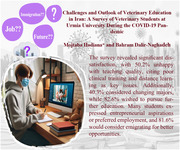

## 1 Introduction

The emergence of modern veterinary education in its current form in Iran dates back more than 85 years ago (1928–1929) when several individuals were sent to France by the government to study veterinary science due to the urgent need of the Ministry of War at that time for controlling diseases and improving horse breeds in the cavalry, as well as combating animal diseases and common diseases between humans and animals. The responsibility for veterinary education was first entrusted to the army. The first veterinary school was founded in 1932–1933 in Tehran, followed by the schools in Shiraz, Urmia and Ahvaz in 1969, 1976 and 1977, respectively (Shahid Chamran University of Ahvaz 2024; Shiraz University 2024; University of Tehran 2024; Urmia University 2024). Currently, according to the National Examination Centre, an affiliated institution of the Ministry of Science that is responsible for conducting annual university entrance exams, there are 21 veterinary schools in Iran, graduating almost 1500 veterinarians annually (The Booklet Guide for University Courses in Iran's National Exam 2021). Some experts suggest that the quality of veterinary education has been sacrificed for quantity. Veterinary education in Iran is primarily pursued at the doctorate level, which comprises six academic years. Each year is divided into two semesters of 16 weeks. This education is offered at two types of universities: state‐run universities and Islamic Azad universities. The latter was founded as a private university in 1982 and headquartered in Tehran and now has hundreds of branches and campuses across the country (Islamic Azad University 2024).

In terms of the domestic animal population, the average livestock population in Iran is as follows: Cattle—5,000,000; sheep—47,000,000; goats—17,000,000 and camels—150,000 (Kiani et al. 2021; Mirahmadi et al. 2022). However, it is important to note that these statistics are approximations, as there is no accurate data on rangeland‐dependent livestock in Iran. Furthermore, there is no precise data on the exact number of pets in Iran, primarily due to the absence of a comprehensive pet registration system in the country. Nevertheless, unofficial data suggest that there are around 7–8 million pets (mainly dogs and cats) in Iran. Although dogs in Iran in the past, especially in rural areas, were mainly kept for utilitarian purposes, the current form of their ownership as pets is almost a new phenomenon. It has been on the rise, particularly in urban areas, as more people have become interested in keeping pets as companions and for their emotional and social benefits. Keeping dogs as pets in Iran, a predominantly Muslim country, may pose challenges and is frowned upon by certain segments of society who adhere to some religious teachings (Keeping Dogs in the Living Environment 2019).

After finishing secondary school, Iranian students primarily take the University National Exam known as the ‘Concur’ and compete in three main disciplines: humanities, mathematics and physics and natural sciences. The top three fields within Natural Sciences that most students aspire to enter are medicine, dentistry and pharmacy. Consequently, it is presumed that most veterinary students initially aim for these three fields, but due to their performance in the university entrance exam, they are redirected to veterinary studies (The Islamic Republic News Agency 2019).

The emergence of COVID‐19 in 2019 completely transformed the world, including schools and universities. Lockdowns were enforced and distance learning became a new concept in education. University students who previously lived on campuses and attended lectures and practicals were forced to return home and follow lessons through their computer monitors. Given these circumstances and the context mentioned above, we conducted a survey among veterinary students at Urmia University in Iran. The aim of the survey was to explore the satisfaction and outlook of veterinary students towards their discipline. In addition, we sought to identify the current challenges that students are facing at the moment.

## 2 Materials and Methods

The survey was voluntary and anonymous, and the participants were veterinary students enrolled at the Faculty of Veterinary Medicine at Urmia University. The questionnaire was designed online to explore the career preferences and opinions of students regarding various aspects of their courses, including the quality of teaching and their future outlook. The link to the questionnaire was sent to all veterinary students via email along with an attached consent form that explained the purpose of the study. The questionnaire was available online for approximately 1 month, from 20 April to 20 May 2021. Out of the 292 students on the faculty, 201 of them completed the survey.

Statistical analysis of data was performed with the SPSS statistical package, version 27 for Windows (Chicago, IL, USA). The descriptive analysis of the data was carried out by calculating the percentage of total responses. The qualitative text data were subjected to thematic analysis, which involved identifying and categorising common themes in the responses. Each answer was then classified and listed according to these themes (Kuckartz 2019).

## 3 Results

**FIGURE 1:**
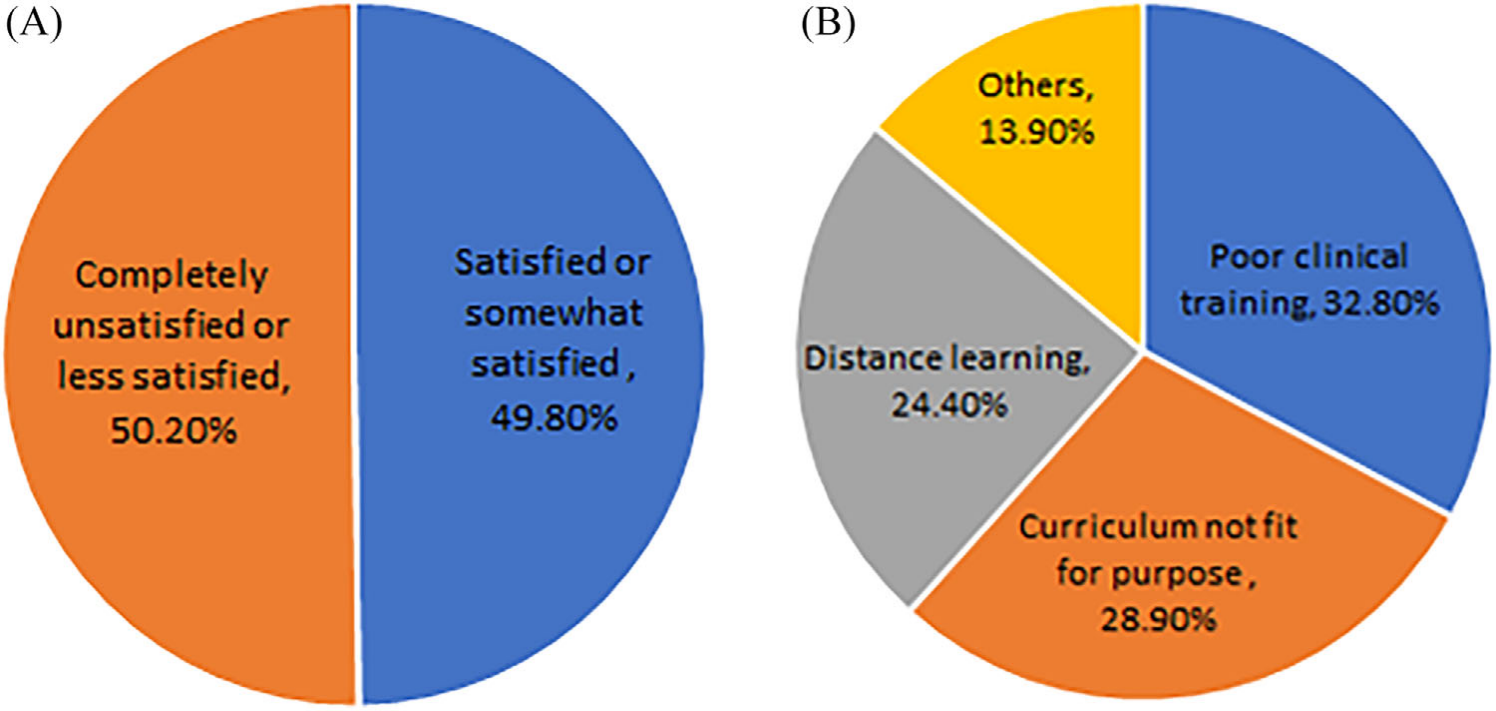
(A) Students' opinion on the quality of their teaching and (B) the reasons for students' dissatisfaction.

Out of a total of 201 participants in the study, 36.8% were male and 63.2% were female. Slightly over 49% of the respondents were either very satisfied or somewhat satisfied with the quality of the teaching, whereas 50.2% were completely unsatisfied or less satisfied. As shown in Figure 1A,B, the main reasons for this dissatisfaction were poor clinical training (32.8%), a curriculum not fit for the purpose (28.9%) and distance learning (24.4%). When asked if they would change their major if given the opportunity, 40.3% of the students answered ‘yes’, with the majority (90.8%) choosing medicine, dentistry and pharmacy.

**FIGURE 2:**
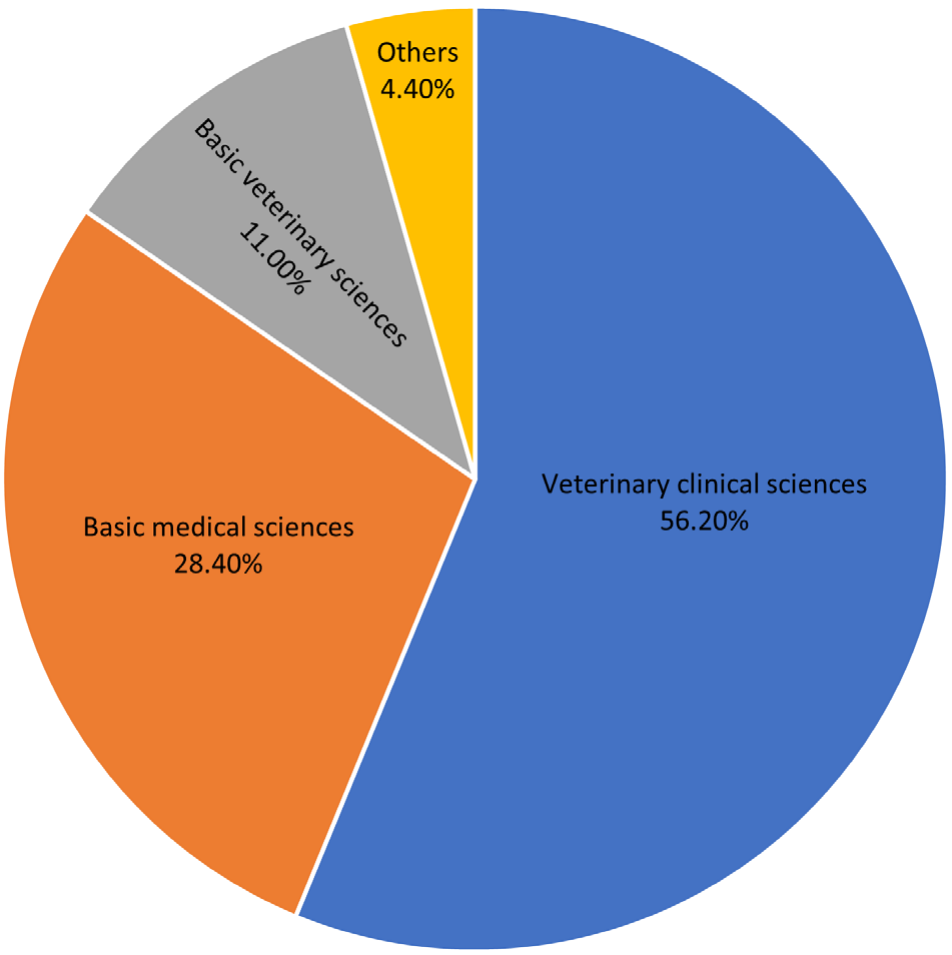
The postgraduate courses in which students showed an interest in studying after completing their undergraduate degrees.

In another part of the survey, 82.6% of students declared that they would continue their education after finishing their undergraduate course. Veterinary clinical sciences (56.2%), basic medical sciences (28.4%) and basic veterinary sciences (11%) were among the most favourite postgraduate courses they would prefer to attend (Figure 2).

**FIGURE 3:**
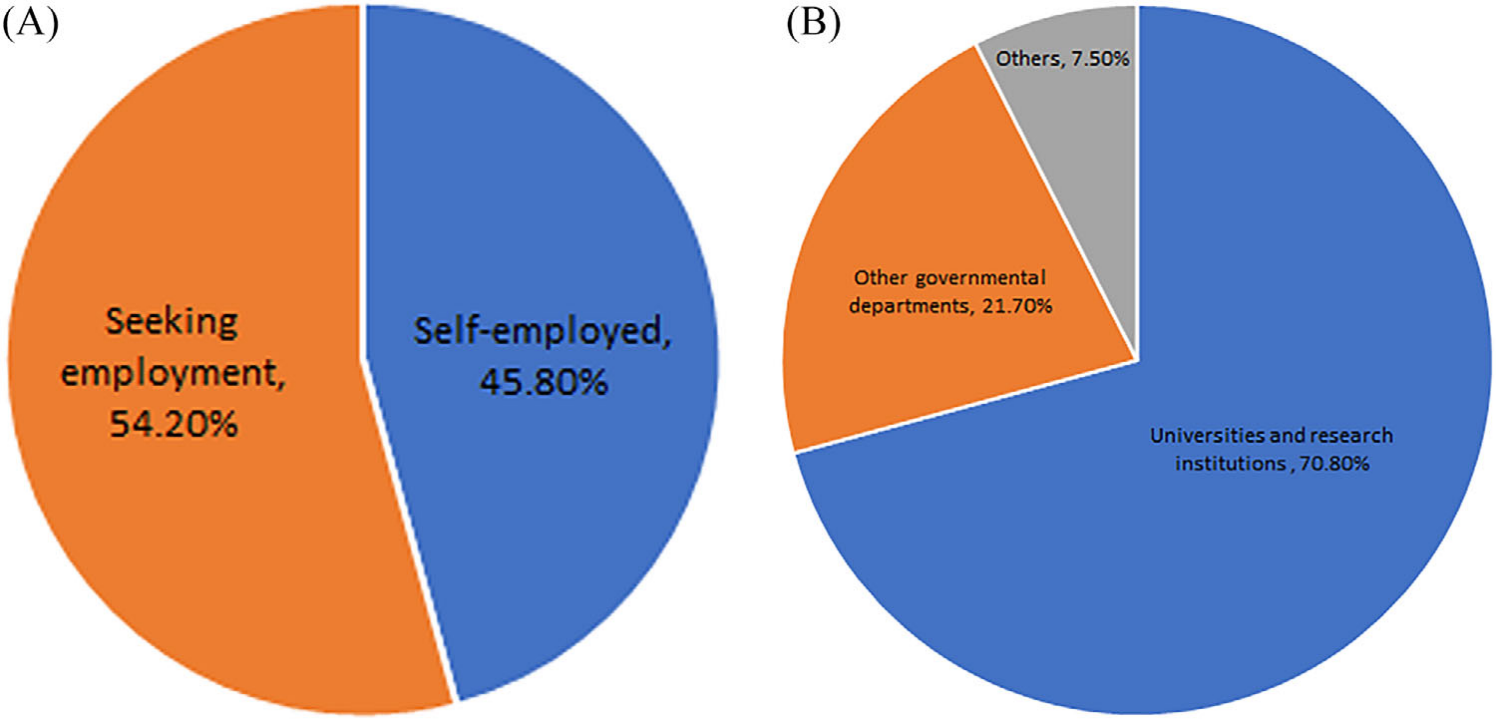
(A) Students' willingness to start their own business or be employed and (B) preference for employment in other sectors.

Regarding their future profession, 45.8% of the participants expressed a desire to start their own business or practice. On the other hand, 54.2% preferred employment opportunities in universities and research institutions (70.8%) as well as other governmental departments (21.7%) (see Figure 3A,B). Interestingly, 81.6% of the participants mentioned they would consider immigration to pursue their career prospects, whereas only 18.4% firmly stated their intention to remain in Iran.

**FIGURE 4:**
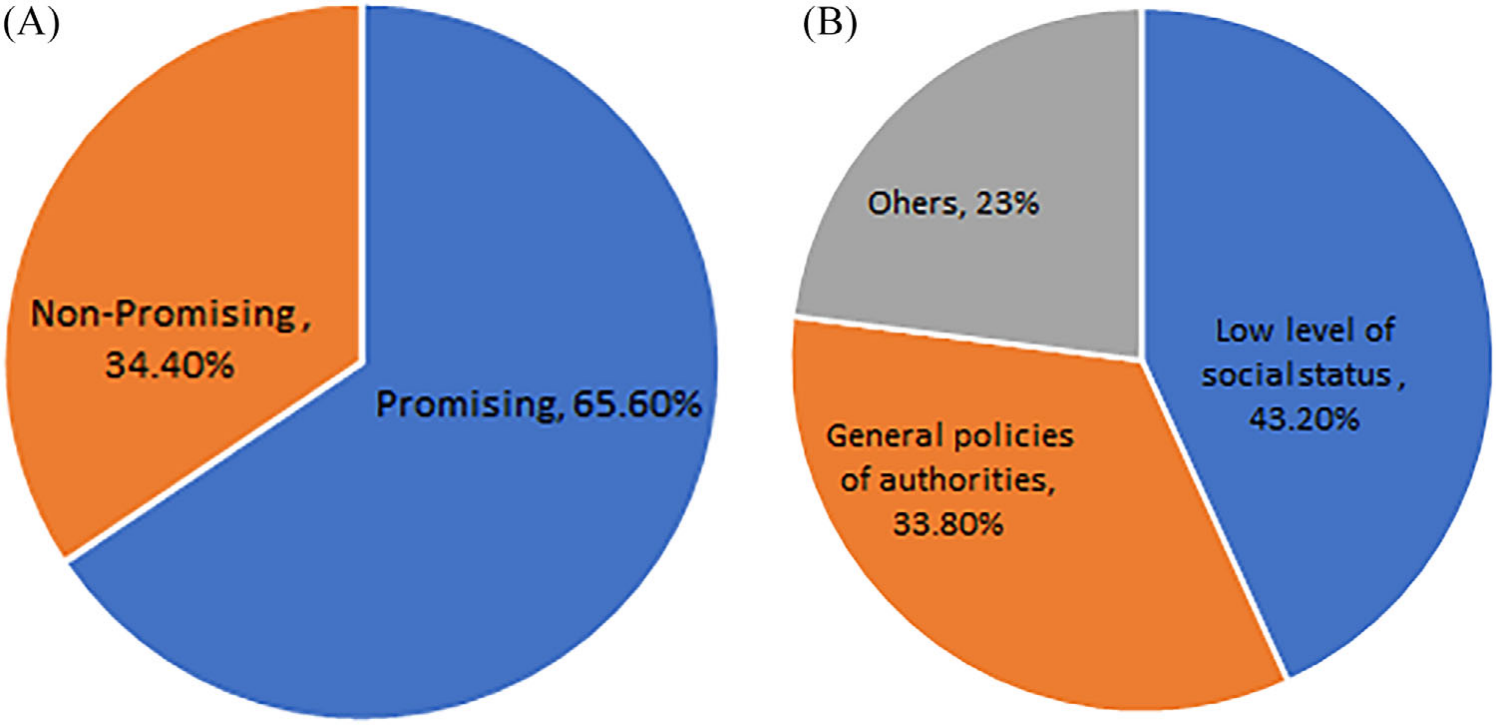
(A) Students' outlook towards veterinary medicine in Iran and (B) the reasons for the backwardness of veterinary medicine in the country.

When participants were asked about their expectations for their future income level, 55.3% expressed satisfaction with it, whereas 44.7% believed that the money would not align with the time and effort they have invested in their studies. A total of 65.6% of the respondents were of the opinion that there would be a bright and promising future for veterinary medicine in Iran, whereas 34.4% believed the complete opposite. When students were asked about the reasons for the backwardness of veterinary medicine in Iran, they mainly blamed the low level of social status (43.2%) and the general policies of authorities (33.8%) (see Figure 4A,B). Finally, when the students were asked whether they would recommend studying veterinary medicine to others, only 21.9% of them answered affirmatively.

## 4 Discussion

In the current study, our goal was to assess the satisfaction and perspective of veterinary students regarding their course and future. Modern veterinary medicine and its education are relatively new concepts in Iran. In the past, like almost all other societies, animals were generally seen as subordinate to humans and were only viewed as tools for human convenience (Cucchi and Arbuckle 2021). In Islam, humans are generally considered the most honourable creation and the Holy Quran emphasises the creation of all things on earth for human benefit (The Holy Qur'an, 2,29, Khattab n.d.). However, there are numerous quotes from the prophet Muhammad that condemn cruelty towards animals and promote kindness towards them (Abdul Rahman 2017). The recent trend in the West, which places a high value on animal welfare and considers companion animals as true family members, is relatively new in Iran and is sometimes scorned by by conservatives. Within this context, it is not surprising that medicine is highly respected and culturally preferred over veterinary care in Iranian society. Furthermore, the relatively higher earnings of physicians compared to veterinarians often lead parents to push their children to pursue a career in medicine (Nejat, Emami Razavi, and Rashidian 2006).

Under these circumstances, it is likely that not all veterinary students can be stratified by their chosen discipline. A study conducted in six universities in Iran using Holland's Vocational Typology and Personality questionnaire on first‐year veterinary students revealed that the dominant personality type in the veterinary environment was realistic, investigative and social and the compatibility of the personality types of the studied students was as follows: 50.09% had complete compatibility, 17.36% had moderate compatibility, 12.81% had low compatibility and 10.74% were a mismatch with the field of veterinary medicine (Bahonar, Chalabi, and Mostafavi 2011). In our study, we did not differentiate students based on their semesters, and as mentioned, 40.3% of respondents would choose to change their course if given a second opportunity. Comparing our results with Bahonar's study, which was based on Holland's questionnaire, shows that the interest of veterinary students remained relatively consistent throughout their studies. Although the Holland questionnaire of typology can provide insight into a person's interests and potential career paths, it is not a definitive guide to career success. There are numerous factors, such as passion, adaptability, support and mentorship, environment, experience and job flexibility, that can help people succeed in a career or course that does not match their typology (Haenggli and Hirschi 2020; Moake, Dougherty, and Dreher 2023). Unfortunately, it appears that these factors are currently missing in Iran's veterinary education system. As revealed in our study, more than half of the respondents were dissatisfied with the quality of teaching. Although the study was conducted during the COVID‐19 pandemic and 24.4% blamed distance learning for this dissatisfaction, there were other reasons as well. These included poor clinical training and a curriculum not fit for the purpose. Currently, under a 6‐year veterinary education program, veterinary students spend over 4 years gathering information on almost every aspect of veterinary with little value for the real needs of a veterinary surgeon. They then enter clinical training that is less equipped technologically, where they also find previous knowledge to be less useful.

In our study, nearly a quarter of students expressed pessimism about the future of veterinary medicine in Iran. They identified two main factors contributing to this sentiment: the *low social status of the occupation and the misguided policies implemented* by authorities. These factors can be further explained by the relatively high unemployment rate among veterinary graduates. According to a report from the Statistical Centre of Iran, the unemployment rate for veterinary graduates in 2018 was 13.3%, slightly higher than the overall unemployment rate of 12.1.% for the active labour force (Heidarnejad and Ghaemmaghami 2023; Summary of the Results of the Workforce Statistics 2024).

According to Iran's Sixth Development Program, it had been proposed that the country would need a total of 18,732 veterinarians, including those in the public and private sectors, by the Year 2025. However, there are currently over 24,000 registered veterinarians in the country, which clearly exceeds the country's needs. In addition, one could add 3000–4000 veterinarians to that figure who do not practice and, therefore, do not feel the need to register with the National Veterinary Council. These veterinarians mainly work in other institutions. As the surveyed students rightly pointed out, this situation arises from an erroneous policy in the higher education system that allows a high number of students to enrol in 21 veterinary faculties or colleges, whereas 40 years ago, there were only four. For example, in the academic year 2020–2021, there were 1286 new entrants (The Booklet Guide for University Courses in Iran's National Exam 2021). The alarming rate of unemployment among veterinary graduates, which is also fairly common among other university graduates, has led various veterinary associations and societies to launch campaigns and petition authorities such as the president and the parliament on this issue. Unemployment has forced some veterinary graduates to seek employment in unrelated fields, pursue postgraduate courses to enhance their qualifications for better job prospects or consider relocating abroad. Our study has also revealed that 82.6% of respondents expressed a desire to continue further studies, whereas a significant and considerable number contemplated immigration. The number of Iranian students studying abroad increased to 56,376 in 2018, which was a 3.2‐fold increase compared to 2000, when the population was only 17,447. This placed Iran at the rank of 19th among countries that send students abroad. Although the desire to study at top universities worldwide can be a major motivation for Iranian students, economic factors and the opportunity for a better life abroad through education are also significant factors contributing to the increasing trend of Iranian student's migration. In winter 2021, a survey was conducted to assess the tendencies and decisions of Iranian students and graduates after 2013. The survey findings revealed that the overall status of the country and economic considerations were the primary motivations for students and graduates to migrate. The lack of opportunities to make a meaningful impact and the feeling of being useless in the country further strengthen the motivations for migration, leading many students and graduates to consider leaving. The top five destinations for Iranian international students in 2018 were the United States, Turkey, Germany, Italy and Canada. In Canada, for example, Iranian students accounted for a population of 4535, making up almost 2% of the international student market. This represented an approximately 17% increase from the previous year (2017), when the number was 3884 (Iran Migration Observatory 2024).

One of the major reasons for dissatisfaction among veterinary students in our survey about their course was distance learning. Virtual or online learning became wildly known when the World Health Organisation designated COVID‐19 a global health emergency on 30 January 2020 and a pandemic on 11 March 2020. As a result, lockdowns were imposed and almost all classroom teaching activities were shut down. Students' residential houses were closed, and students were sent home. It was a challenging time for the worldwide education system (Sadeghinezhad 2023). The transformation from classrooms to e‐learning, even in developed countries with advanced platforms, was not carried out smoothly. However, the impact of the lockdown was even more severe in many developing countries due to a lack of preparedness and infrastructure for online learning (Aminul Islam and Shah Alam 2021; El Mourabit et al. 2023). Historically, veterinary education has heavily relied on face‐to‐face teaching, practicals, hands‐on workplace learning and clinical training. Although distance learning during COVID‐19 affected education, it appears that some courses, such as veterinary medicine, have been more affected. A study on veterinary students from 92 countries demonstrated that over 95% of students were of the opinion that their studies had been adversely affected to some extent; with 47.5% believing the impact was significant (Routh et al. 2021). The American Veterinary Medical Association (AVMA) also surveyed 2000 practice owners and concluded that SARS‐CoV‐2 had disastrous effects on veterinary clinical practices (AVMA 2024). Another study conducted by Sadeghinezhad on the impact of online veterinary anatomy education during COVID‐19 in Iran found that only 0.9% of students considered online education suitable for the practical part of anatomy. In addition, half of the participants (50.1%) also believed that e‐learning was not suitable for any of the theoretical and practical aspects (Sadeghinezhad 2023). Another study in Iraq among veterinary students revealed that 34.76% and 47.10% of respondents were of the opinion that distance learning has significantly affected theoretical and practical lessons, respectively (Al‐Salihi, Jihad, and Saleh 2024). During a study conducted in Turkey amidst the pandemic, it was found that up to 70% of veterinary students anticipated in‐person catch‐up programs for the same lessons that had been delivered online (Aslım, Tekindal, and Yiğit 2023). These findings aligned with the results of our own study, where a significant number of respondents blamed online education as one of the main sources of their dissatisfaction.

The COVID‐19 pandemic has posed numerous challenges for higher education. On the one hand, distance learning has created new possibilities for more flexible and dynamic teaching methods, utilising advanced technological tools to deliver a wide array of educational materials. In addition, it has the potential to reduce the costs associated with veterinary studies. However, on the other hand, distance learning has proven to be difficult and almost impractical for teaching hands‐on practical and clinical skills, leading to dissatisfaction among students. Compounding this issue is the lack of adequate infrastructure and poor internet access in some developing countries, exacerbating the problem further.

## 5 Conclusion

In conclusion, the study conducted among veterinary students at Urmia University revealed several challenges in the veterinary education system in Iran. These challenges mainly included the quality and content of teaching from various perspectives, high unemployment among graduates and the low social status of veterinary medicine in society. A considerable number of students also expressed a desire to change their major to medicine, dentistry or pharmacy if given the opportunity. Despite these challenges, the majority of students expressed a desire to continue their education after completing their undergraduate course, with veterinary clinical sciences being the most popular postgraduate option. When it comes to future employment, students were almost evenly divided between a preference for starting their own business or practice or seeking employment by governmental and non‐governmental institutions. Interestingly, a high percentage of participants mentioned they would consider emigrating to pursue their career prospects, highlighting the high unemployment rates among veterinary graduates in Iran. Overall, the study emphasised the need for improvements in teaching quality. This can be achieved through the design of a new curriculum that is fit for the purpose and by providing better clinical training. It also suggested that authorities should adopt new policies, such as limiting the quota for veterinary student admissions across the country. In addition, creating more employment opportunities in the veterinary field in Iran was identified as a crucial step. Finally, the study highlighted the negative impact of the COVID‐19 pandemic on veterinary education. Although distance learning can be used as a supplementary tool, it cannot substitute practice‐orientated education in veterinary medicine.

## Author Contributions

Mojtaba Hadian and Bahram Dalir‐Naghadeh performed the conceptualisation. Mojtaba Hadian and Bahram Dalir‐Naghadeh created the methodology, investigation and data analysis. Mojtaba Hadian wrote the original draft. Mojtaba Hadian and Bahram Dalir‐Naghadeh did reviewing and editing work. All authors have read and agreed to the published version of the manuscript.

## Ethics Statement

The study was conducted according to the guidelines approved by the Ethics Committee of Urmia University (IR‐UU‐AEC‐3/78; 2021).

## Consent

Informed consent was obtained from all subjects involved in the study.

## Conflicts of Interest

The authors declare no conflicts of interest.

### Peer Review

The peer review history for this article is available at https://www.webofscience.com/api/gateway/wos/peer-review/10.1002/vms3.70081.

## Data Availability

Data are available upon request to the authors.
